# Serotype distribution and antimicrobial resistance of Streptococcus pneumoniae in paediatric patients in Japan (2020–2023)

**DOI:** 10.1099/jmm.0.002105

**Published:** 2025-12-12

**Authors:** Satoshi Nakano, Takao Fujisawa, Shota Koide, Yo Sugawara, Bin Chang, Yutaka Ito, Shigeru Suga, Makoto Ohnishi, Yukihiro Akeda, Motoyuki Sugai

**Affiliations:** 1 Antimicrobial Resistance Research Center, National Institute of Infectious Diseases, Japan Institute for Health Security, Tokyo, Japan; 2 National Hospital Organization, Mie National Hospital, Tsu, Japan; 3 Department of Bacteriology I, National Institute of Infectious Diseases, Japan Institute for Health Security, Tokyo, Japan; 4 Nagoya City University, Graduate School of Medical Science, Nagoya, Japan

**Keywords:** invasive pneumococcal diseases, Japan, paediatric, pneumococcal conjugate vaccines, *Streptococcus pneumoniae*, whole-genome sequencing

## Abstract

**Introduction.** *Streptococcus pneumoniae* remains a major pathogen causing invasive diseases in children worldwide. Although pneumococcal conjugate vaccines (PCVs) have significantly reduced the disease burden, non-vaccine serotypes and antimicrobial resistance continue to be of concern.

**Hypothesis/ Gap Statement.** The epidemiology of paediatric invasive pneumococcal disease (IPD) and antimicrobial resistance patterns in Japan following the coronavirus disease 2019 pandemic and prior to the introduction of PCV15 and PCV20 has not been fully characterized.

**Aim.** To investigate the recent distribution of pneumococcal serotypes, antimicrobial susceptibility and genetic characteristics of isolates derived from paediatric patients in Japan from 2020 to 2023.

**Methodology.** We conducted a nationwide, prospective surveillance study from March 2020 to April 2023. A total of 151 pneumococcal isolates (126 from IPD cases and 25 from non-IPD cases) were collected from children under 15 years of age. Serotyping, antimicrobial susceptibility testing and whole-genome sequencing were performed to assess epidemiological and genomic features.

**Results.** No patient mortality was reported, but sequelae were observed in 4 (3.2%) of 125 IPD patients. The most common serotypes in IPD were 15B/C (23.0%), 15A (11.1%) and 24B (10.3%). Among 126 IPD isolates, the vaccine coverage rates for PCV13, 15 and 20 were 0.8, 13.5 and 42.1%, respectively. Overall resistance rates to penicillin (PEN), cefotaxime, meropenem (MEM) and erythromycin (ERY) were 31.8, 15.9, 18.5 and 88.7%, respectively. Serotypes 15A-CC63 and 35B-CC558 showed high resistance rates to *β*-lactams, including MEM. Genomic analysis revealed that the predominant genotypes were 15B/C-CC199, 15A-CC63, 24B-CC2754 and 10A-CC5236.

**Conclusion.** Non-vaccine and PEN-, MEM- and ERY-resistant clones, particularly 15A-CC63 and 35B-CC558, were prevalent among paediatric pneumococci in Japan. Even with PCV20, less than half of the IPD isolates were covered; this underscores the need for ongoing genomic surveillance, antimicrobial stewardship and consideration of expanded-valency vaccines targeting additional serotypes, such as 15A and 35B.

## Introduction

*Streptococcus pneumoniae* is the major pathogen responsible for community-acquired pneumonia, bacterial meningitis, otitis media and other illnesses [1]. Pneumococcal conjugate vaccines (PCVs) have been widely adopted as part of routine immunization programmes in many countries since the early 2000s to prevent invasive pneumococcal diseases (IPDs) in children, particularly infants and toddlers. However, a previous study estimated that ~294,000 deaths attributed to pneumococcal infection occurred in human immunodeficiency virus-uninfected children aged 1–59 months in 2015 [2]. With the onset of the COVID-19 pandemic, the incidence of pneumococcal disease temporarily declined across many countries [3,4], making it difficult to accurately estimate the disease burden during that period. Nevertheless, in 2022, more than 55,000 children aged 0–4 years died from pneumococcal disease in India [5]. Therefore, IPD in children remains an important public health concern. Moreover, it causes invasive infections in adults, with a disease incidence rate of 5.40 per 100,000 population [6] and healthcare-associated costs of up to $47,489 in healthy and high-risk adults in the USA in 2019 [7].

In Japan, PCV7 was introduced as an optional vaccine for children in 2011 and became part of the routine vaccination schedule in 2013. Subsequently, in the same year, it was replaced by PCV13 and continued to be used until March 2024. Eventually, it was replaced by PCV15 in April 2024. The vaccination programme followed a 3+1 schedule, and vaccination coverage exceeded 98% in 2018. However, despite the high vaccine uptake, shifts in serotype distribution and antimicrobial resistance became evident. For instance, as reported by Suga *et al.* [8], PCV7 introduction led to a 57% reduction in the incidence of IPD; however, the proportion of meropenem (MEM) resistant isolates significantly increased in serotypes 19A and 15A after the implementation of PCV7.

We previously conducted a nationwide surveillance study of paediatric invasive and non-IPDs in Japan between 2012 and 2017 [9,10]. We demonstrated an increase in MEM-resistant pneumococci during the first half of our study period (2012–2014). In the latter half (2015–2017), the MEM-resistance rates exceeded 10% in IPD strains and 30% in non-IPD strains. Following the introduction of PCV7, the prevalence of serotypes 19A and 15A increased, much of which was associated with antimicrobial resistance. Of note, similar to many other countries that introduced PCVs, there was an increase in both IPD and non-IPD cases attributable to serotype 19A after the introduction of PCV7 [11,15], which subsequently decreased after switching to PCV13. Furthermore, after the introduction of PCV13, an increase in the prevalence of serotypes 24B/F, 15A and 12F in IPD cases was observed. However, the trends in serotype epidemiology following the onset of the COVID-19 pandemic were not fully elucidated.

According to previous studies demonstrating an increase in MEM-resistant pneumococci in Japan, the predominant resistant clones are serotypes 15A-CC63, 19A-CC3111 and 35B-CC558. Of particular concern are serotypes 15A-CC63 and 35B-CC558, which exhibited over 70% resistance to MEM [16,17]. Although the association between *β*-lactam resistance in pneumococci and patient outcomes remains controversial, monitoring *β*-lactam resistance trends remains important from a public health perspective to prevent the dissemination of antimicrobial-resistant organisms. The 15A-CC63 and 35B-CC558 isolates had quite distinct penicillin-binding protein (PBP) profiles, indicating that the antimicrobial resistance mechanisms of the two clones are not associated. Conversely, most MEM-resistant 15A-CC63 and 19A-CC3111 isolates possessed *pbp1a*-13, the same allele found in the globally spread MEM-resistant 19A-ST320 isolates [18,20], suggesting that the horizontal transfer of *pbp1a* via recombination events is a potential source of the emergence and spread of MEM-resistant pneumococci. In addition, according to the nationwide antibiogram data of Japanese medical settings published by Japan Nosocomial Infectious Surveillance (JANIS) [21], which was a national surveillance programme organized by the Ministry of Health, Labour and Welfare of Japan, the resistance rate to MEM appeared to increase over the period of the COVID-19 pandemic (15.1% in 2019, 19.1% in 2021 and 20.2% in 2023). Thus, we focused on trends in MEM resistance in pneumococci over the past 15 years.

Considering the impact of the COVID-19 pandemic on pneumococcal epidemiology is important. The incidence of invasive pneumococcal infections in both children and adults declined in many countries, including Japan, during the COVID-19 pandemic [22,25]. The carriage frequency of *S. pneumoniae* in children varies by serotype, and exposure to tobacco smoke as well as contact with other children within the household was reported as a risk factor for carriage [26]. Therefore, lifestyle changes during the COVID-19 pandemic may have influenced the molecular epidemiology of pneumococci by limiting the opportunities for recombination events, which lead to changes in serotype and resistance patterns. Moreover, some studies have reported changes in antimicrobial resistance rates of pneumococci during this period [27,28]. Therefore, various environmental factors may influence the epidemiology of pneumococcal serotypes and the development of antimicrobial resistance. Therefore, obtaining an up-to-date understanding of pneumococcal epidemiology in the post-COVID-19 era to better inform future vaccine policy decisions and clinical practice is necessary.

Accordingly, we conducted a nationwide paediatric surveillance study from 2020 to 2023 to investigate the clinical characteristics of paediatric patients with pneumococcal infections in Japan, pneumococcal serotype distribution, antimicrobial susceptibility profiles and genetic characteristics, such as genotypes and the presence of antimicrobial resistance genes.

## Methods

This was a nationwide, prospective, passive surveillance study of IPD and non-IPD pneumococcal infections. The present study was approved by the Ethics Committee of the National Hospital Organization of Mie National Hospital, Tsu, Japan (approval number 31-101). Informed consent for collecting and using patient information and specimens was obtained from each parent or guardian by the primary physician.

From 2 March 2020 to 30 April 2023, IPD and non-IPD patients were enrolled. The case registration method followed the method used in a previous surveillance study [9]. Briefly, clinicians from medical institutions across the country who diagnosed IPD or non-IPD in patients aged 0–15 years sent the isolates to a central laboratory. IPD was defined as the isolation of pneumococci from the culture of a normal sterile sample. Cases in which *S. pneumoniae* was isolated from sputum and diagnosed as pneumococcal pneumonia or isolated from middle ear effusion and diagnosed as pneumococcal otitis media, but not recovered from sterile specimens, were defined as non-IPD. Basically, a single isolate was submitted for each IPD case. They also completed a questionnaire on patient information and forwarded it to the research office. The isolates received by the central laboratory were analysed using the methods described below.

### Serotyping and antimicrobial susceptibility testing

Serotyping was performed using a pneumococcal antiserum (Statens Serum Institute, Copenhagen, Denmark). The serotype coverage rate was defined as the proportion of isolates belonging to the serotypes included in each PCV. The additional serotype coverage rates of PCV15 and 20 were defined as the percentages of isolates in the population whose serotypes were included in the respective vaccines but not in PCV13. Susceptibility testing for penicillin (PEN), erythromycin (ERY), cefotaxime (CTX) and MEM was performed using the broth microdilution method, following the Clinical and Laboratory Standards Institute (CLSI) guidelines [29]. For the categorization of ‘susceptible’ to antibiotics, we followed the 2023 CLSI recommendation for meningitis isolates; for example, PEN-susceptible was defined as (in mg l^−1^) ≤0.06. For antibiotics in the intermediate resistance category, intermediate resistance was considered part of the resistance category for analysis.

### Whole-genome sequencing analysis

All the isolates collected during the study period were subjected to whole-genome sequencing. Bacterial DNA was extracted using a QIAamp DNA mini kit (Qiagen, Hilden, Germany), and the DNA library for whole-genome sequencing was prepared using an Enzymatics 5×WGS fragmentation mix and WGS ligase reagents (Qiagen). Paired-end sequencing (2×150 bp) was performed on the Illumina HiSeq X FIVE platform (Macrogen Japan Corporation, Tokyo, Japan).

Low-quality reads were trimmed using fastp v0.20.1, followed by genome assembly using Shovill v1.1.0. Multilocus sequence typing [30], PBP typing [31,33] and resistance and pilus gene detection were conducted using an in-house analysis pipeline published previously [34]. In brief, we performed *in silico* Multilocus sequence typing (MLST) using mlst (https://github.com/tseemann/mlst), identified the presence of the *ermB*, *ermTR*, *mefA*, *mefE*, *tetM*, *tetO*, *rrgA-1* (pili1) and *pitB-1* (pili2) genes and searched for mutations within *folA* and *folP* genes by using assembled contigs. GyrA S79F and ParC S79F substitutions were detected. Moreover, we incorporated the ResFinder database into our pipeline to identify other determinants of accessory genome resistance [35]. Clonal complexes (CCs) were defined in agreement with five of the seven loci, with the most predominant sequence type (ST) representing the CC. Global pneumococcal sequence clusters (GPSC) were determined using the PathogenWatch website [36]. After the determination of core genes using Roary v3.13.0 [37] with default parameters, we reconstructed a maximum likelihood tree for the core-genome single nucleotide polymorphisms (SNPs) using raxml-ng v1.1 [38] using the GTR+Γ model.

### Statistical analysis

Logistic regression analysis was performed using R v4.4.1 (R Foundation for Statistical Computing, Vienna, Austria) to evaluate the association between patient age (in months, converted to years) and the prevalence of underlying diseases. Age was modelled as a continuous variable, and the odds ratio (OR) was expressed per 1 year increase. The logit-linearity assumption was assessed by comparing the linear model with a restricted cubic spline model using the likelihood ratio test (LRT). To compare the antimicrobial resistance patterns between IPD and non-IPD isolates, we performed subgroup analyses using Fisher’s exact test. Resistance was defined according to the aforementioned breakpoints. To account for multiple comparisons across different antibiotics, Bonferroni correction was applied, and corrected *P* values<0.05 were considered statistically significant.

## Results

A total of 125 patients with IPD (51 female and 74 male patients) from 54 clinics/hospitals, including 6 university hospitals, and 25 patients with non-IPD (11 female and 14 male patients) from 4 clinics/hospitals were submitted during the study period. The median age in months (first-third quartile) of the patients with IPD was 28 (14–32). In one case (meningitis), two strains with different serotypes (15B and 35B) were detected. This case involved a 5-year-old child with meningitis and a history of bacterial meningitis; however, no information regarding the causative pathogen of prior meningitis or the presence of intracranial anomalies was available in the registry.

Thus, we subsequently tested 151 isolates (126 IPD and 25 non-IPD isolates).

**Fig. 1.**
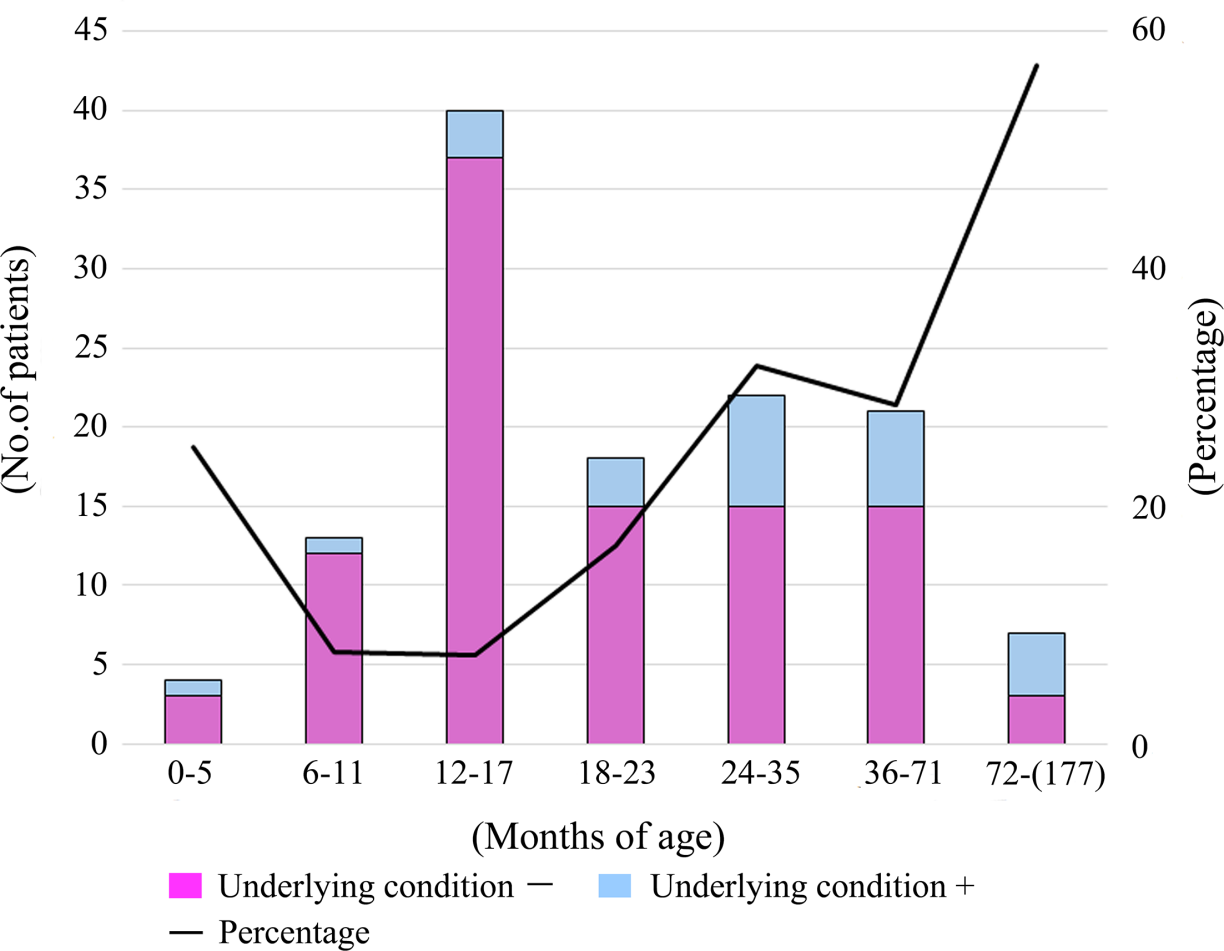
Numbers and rates of patients with underlying diseases among all IPDs in each age group. The prevalence of underlying conditions increased progressively with advancing age group (in months).

Among the 125 patients with IPD, 25 (20.0%) had congenital or malignant underlying diseases (Fig. 1). In univariable logistic regression, increasing age was significantly associated with the presence of underlying diseases (OR per 1 year increase, 1.28; 95% CI: 1.06–1.55; *P*=0.010). Assessment of the logit-linearity assumption revealed no evidence of deviation from linearity (LRT, *P*=0.245; Wald test, *P*=0.125); therefore, age was modelled as a linear term in the main analysis. No cases of patient mortality were observed, but sequelae were noted in four cases (3.2%), of which five isolates were detected, attributable to serotypes 35B-ST558 (*n*=2), 15B/C-ST18738 (*n*=1), 10A-ST1263 (*n*=1) and 15A-ST63 (*n*=1). Among the 125 patients with IPD, 121 (96.8%) received at least one dose of any type (PCV7, 10 or 13) of PCV. All four patients with sequelae received at least one dose of PCV13.

### Serotype distribution

To enhance the readability of the figures, we grouped the patients registered from March 2020 to February 2021 as 2020 patients, those registered from March 2021 to February 2022 as 2021 patients and those registered from March 2022 until the end of the study (April 2023) as 2022 patients.

**Fig. 2.**
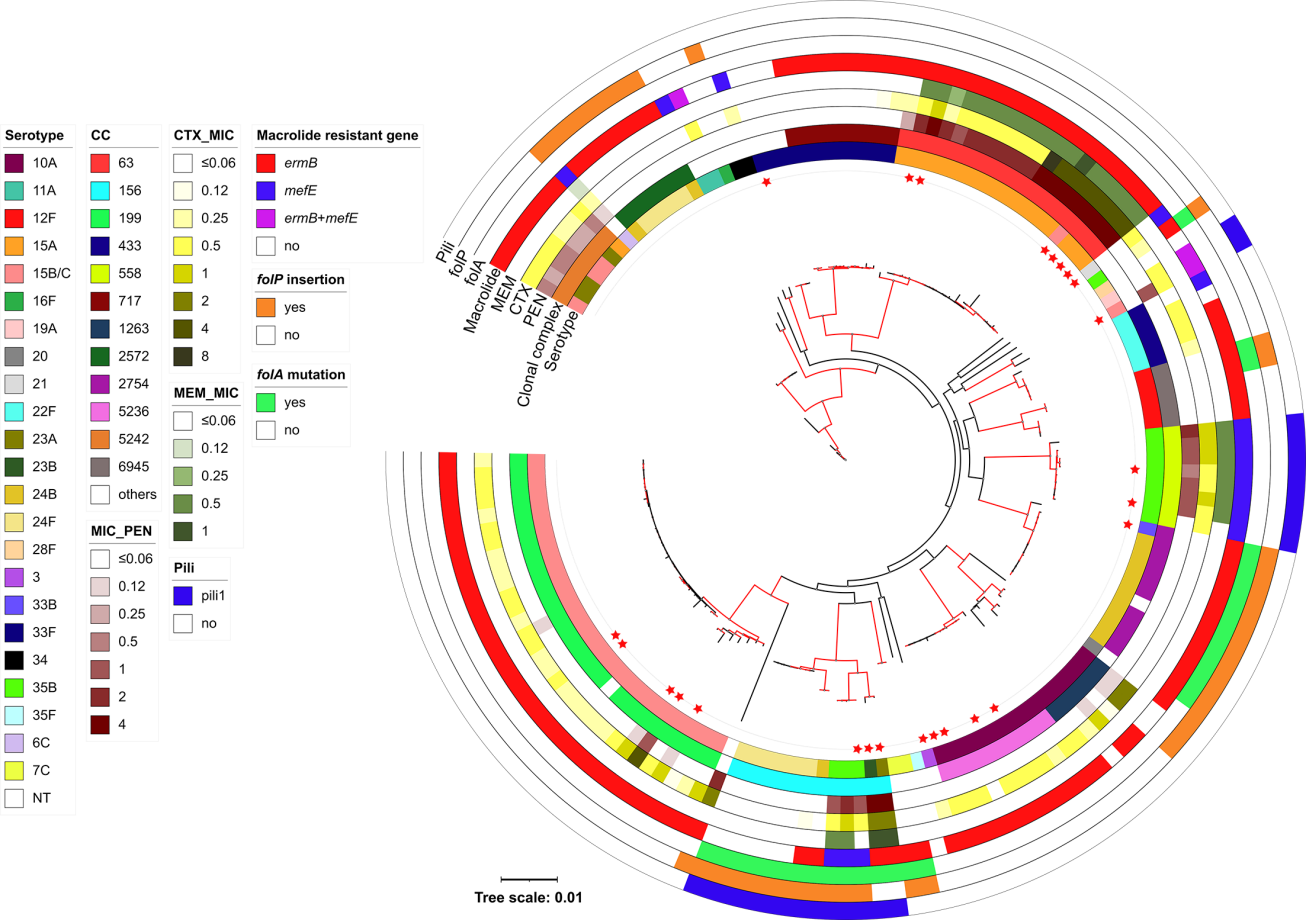
Maximum likelihood tree of all pneumococcal isolates collected between 2020 and 2022 in Japan. Red branches were supported by bootstrap values over 80. Leaves with red stars indicate isolates from non-IPD cases. The outer bands of the phylogenetic tree, from the outside inward, represent the presence/absence of Pili1/2, *folP* insertion, *folA* mutations and macrolide resistance genes (*mefE* and/or *ermB*), the MIC for MEM, CTX and PEN, the CC and the serotype.

Among 126 IPD isolates, the predominant serotypes throughout the study period were 15B (*n*=17, 13.5%), followed by 15A (*n*=14, 11.1%), 24B (*n*=13, 10.3%), 15C (*n*=12, 9.5%), 24F (*n*=12, 9.5%), 10A (*n*=12, 9.5%) and 33F (*n*=11, 8.7%) (Table 1 and Figs.2  3). While the total number of patients was exceptionally low at just 25, even in the non-IPD group, the most predominant serotypes were 15B/C (*n*=6, 24%) and 15A (*n*=6) (Fig. S1, available in the online Supplementary Material). Only one isolate of serotype 19A that was included in PCV13 was detected in a patient with IPD. Among IPD isolates, serotype coverage rates for PCV13, PCV15 and PCV20 were 0.8, 13.5 and 42.1%, respectively. While serotype 24F was the most predominant serotype in patients with IPD in 2020, the incidence of 24F will become extremely rare by 2021 (2020: ten patients, 2021: one patient and 2022: one patient). A similar decreasing trend was observed for serotype 12F (2020: three patients, 2021: two patients and 2022: none).

**Table 1.** Serotype distribution among IPD and non-IPD isolates between 2020 and 2022 in Japan

Serotype	No. (%) of IPD isolates				No. (%) of non-IPD isolates			
	2020 (*n*=43)	2021 (*n*=47)	2022 (*n*=36)	Total (*n*=126)	2020 (*n*=9)	2021 (*n*=10)	2022 (*n*=6)	Total (*n*=25)
PCV13 serotypes								
19A	1 (2.3)	0 (0)	0 (0)	1 (0.8)	0 (0)	0 (0)	0 (0)	0 (0)
3	0 (0)	0 (0)	0 (0)	0 (0)	0 (0)	1 (10)	0 (0)	1 (4)
Total of PCV13 serotypes	1 (2.3)	0 (0)	0 (0)	1 (0.8)	0 (0)	1 (10)	0 (0)	1 (4)
PCV15 serotypes								
22F	2 (4.7)	2 (4.3)	1 (2.8)	5 (4.0)	0 (0)	0 (0)	0 (0)	0 (0)
33F	4 (9.3)	5 (10.6)	2 (5.6)	11 (8.7)	1 (11.1)	0 (0)	0 (0)	1 (4)
Total of additional PCV15 serotypes	6 (14.0)	7 (14.9)	3 (8.3)	16 (12.7)	1 (11.1)	0 (0)	0 (0)	1 (4)
Total of PCV15 serotypes	7 (16.3)	7 (14.9)	3 (8.3)	17 (13.5)	1 (11.1)	1 (10)	0 (0)	2 (8)
PCV20 serotypes								
10A	0 (0)	7 (14.9)	5 (13.9)	12 (9.5)	1 (11.1)	2 (20)	1 (16.7)	4 (16)
11A	1 (2.3)	1 (2.1)	0 (0)	2 (1.6)	0 (0)	0 (0)	0 (0)	0 (0)
12F	3 (7.0)	2 (4.3)	0 (0)	5 (4.0)	0 (0)	0 (0)	0 (0)	0 (0)
15B	1 (2.3)	7 (14.9)	9 (25)	17 (13.5)	1 (11.1)	0 (0)	1 (16.7)	2 (8)
Total of additional PCV20 serotypes	5 (11.6)	17 (36.2)	14 (38.9)	36 (28.6)	2 (22.2)	2 (20)	2 (33.3)	6 (24)
Total of PCV20 serotypes	12 (27.9)	24 (51.1)	17 (47.2)	53 (42.1)	3 (33.3)	3 (30)	2 (33.3)	8 (32)
Non-PCV20 serotypes								
15A	4 (9.3)	5 (10.6)	5 (13.9)	14 (11.1)	3 (33.3)	2 (20)	1 (16.7)	6 (24)
15C	4 (9.3)	7 (14.9)	1 (2.8)	12 (9.5)	1 (11.1)	3 (30)	0 (0)	4 (16)
16F	0 (0)	1 (2.1)	0 (0)	1 (0.8)	0 (0)	0 (0)	0 (0)	0 (0)
20	0 (0)	0 (0)	1 (2.8)	1 (0.8)	0 (0)	0 (0)	0 (0)	0 (0)
21	0 (0)	0 (0)	0 (0)	0 (0)	1 (11.1)	0 (0)	0 (0)	1 (4)
23A	2 (4.7)	0 (0)	1 (2.8)	3 (2.4)	1 (11.1)	0 (0)	0 (0)	1 (4)
23B	0 (0)	0 (0)	0 (0)	0 (0)	0 (0)	1 (10)	0 (0)	1 (4)
24B	7 (16.3)	2 (4.3)	4 (11.1)	13 (10.3)	0 (0)	0 (0)	0 (0)	0 (0)
24F	10 (23.3)	1 (2.1)	1 (2.8)	12 (17.5)	0 (0)	0 (0)	0 (0)	0 (0)
28F	0 (0)	1 (2.1)	0 (0)	1 (0.8)	0 (0)	0 (0)	0 (0)	0 (0)
33B	0 (0)	0 (0)	0 (0)	0 (0)	0 (0)	0 (0)	1 (16.7)	1 (4)
34	0 (0)	1 (2.1)	1 (2.8)	2 (1.6)	0 (0)	0 (0)	0 (0)	1 (4)
35B	0 (0)	5 (10.6)	4 (11.1)	9 (7.1)	0 (0)	1 (10)	2 (33.3)	3 (12)
35F	0 (0)	0 (0)	1 (2.8)	1 (0.8)	0 (0)	0 (0)	0 (0)	0 (0)
6C	1 (2.3)	0 (0)	0 (0)	1 (0.8)	0 (0)	0 (0)	0 (0)	0 (0)
7C	2 (4.7)	0 (0)	0 (0)	2 (1.6)	0 (0)	0 (0)	0 (0)	0 (0)
nt	1 (2.3)	0 (0)	0 (0)	1 (0.8)	0 (0)	0 (0)	0 (0)	0 (0)
Total of non-PCV20 serotypes	31 (72.1)	23 (48.9)	19 (52.8)	73 (57.9)	6 (66.7)	7 (70)	4 (66.7)	17 (68)

NT, Non-typeable.

**Fig. 3.**
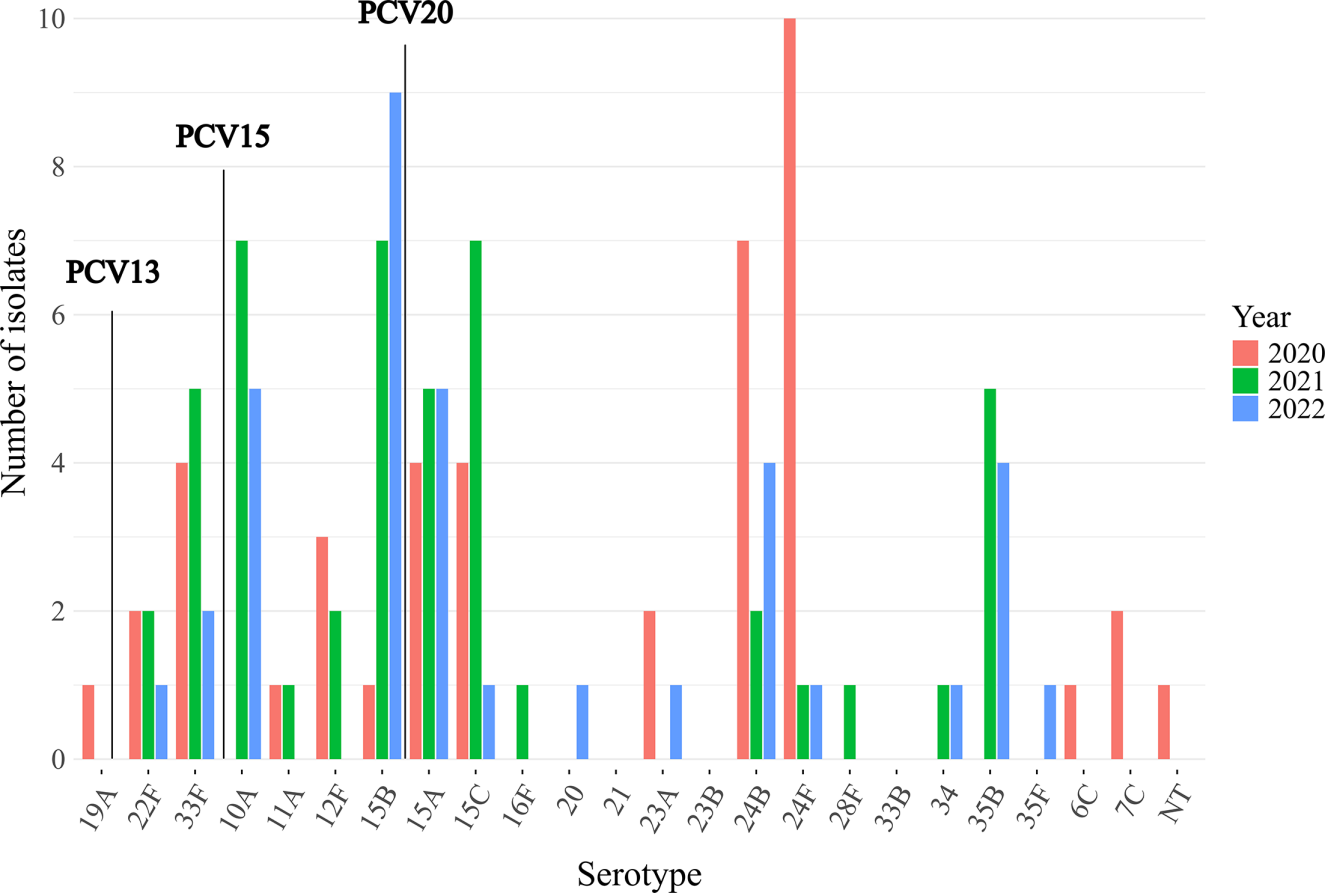
The number of isolates for each serotype obtained from IPD patients in 2020, 2021 and 2022 in this study. Among the total number of IPD isolates, the proportions of isolates covered by PCV13, PCV15 and PCV20 were 0.8, 13.5 and 42.1%, respectively.

### Antimicrobial susceptibility

**Fig. 4.**
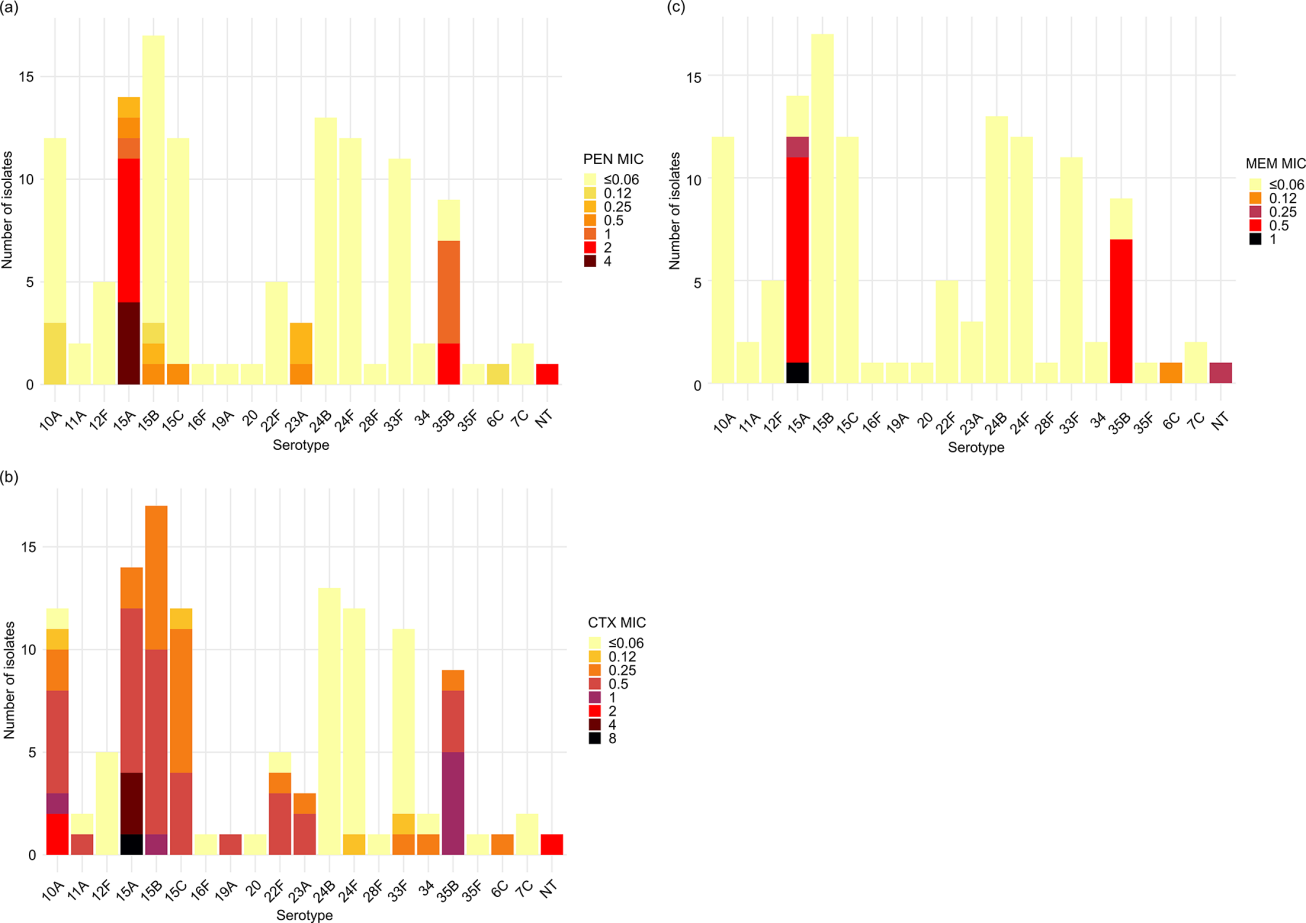
MIC distribution of PEN, CTX and MEM in each pneumococcal serotype in Japan. According to the CLSI criteria, isolates are interpreted as susceptible at MICs≤0.06 µg ml^−1^ for PEN (meningitis), ≤0.5 µg ml^−1^ for CTX and ≤0.25 µg ml^−1^ for MEM. (**a**) PEN, (**b**) CTX and (**c**) MEM.

Among 126 IPD isolates, PEN, CTX, MEM and ERY resistance rates (defined as PEN MIC≥0.12, CTX MIC≥1.0, MEM MIC≥0.5 and ERY MIC≥0.5 mg l^−1^) were 26.2, 11.1, 14.3 and 87.3%, respectively. Among 25 non-IPD isolates, PEN, CTX, MEM and ERY resistance rates were 60.0, 40.0, 40.0 and 96.0%, respectively (Figs 4, S2 and S3 and Table S1). When resistance rates were further compared between IPD and non-IPD cases, resistance to PEN, CTX and MEM was more frequently detected among isolates from IPD, with Bonferroni-corrected *P* values of 0.0071, 0.0047 and 0.034, respectively, whereas ERY resistance remained uniformly high in both groups (*P*=1.0 after Bonferroni correction) (Table S1).

When examining the drug resistance rates of all 151 isolates for each serotype with 10 or more isolated strains [serotype 15B/C (*n*=35), 15A (*n*=20), 10A (*n*=16), 24B (*n*=13), 24F (*n*=12), 33F (*n*=11) and 35B (*n*=11)], serotypes 15A and 35B exhibited high resistance rates for PEN (100%, 90.9%), CTX (45.0%, 45.5%) and MEM (85.0%, 81.8%), respectively.

### Genetic characteristics

We detected 36 combinations of serotypes and CC (genotypes) (Fig. 2 and Table S2). The predominant genotypes were 15B/C-CC199 (*n*=30), 15A-CC63 (*n*=19), 24B-CC2754 (*n*=10) and 10A-CC5236 (*n*=10). When considering only 126 IPD strains, the top 3 genotypes remained the same as those mentioned above [15B/C-CC199 (*n*=25), 15A-CC63 (*n*=13) and 24B-CC2754 (*n*=10)]; however, the fourth most common genotype was 33F-CC717 (*n*=9).

For the predominant clone serotypes 15B/C-CC199 and 15A-CC63, the typical genomic characteristics included *ermB* (+), *tetM* (+), *folA* mutation (−), *folP* insertion (−) and Pilus 1/2 (−) (Table S2).

Among the 134 ERY-resistant isolates, 115 (85.8%) harboured only *ermB*, 16 (11.9%) harboured only *mefE* and 3 (2.2%) harboured both *ermB* and *mefE* (Fig. S4). All 118 isolates with *ermB* had *tetM*, whereas 5 (31.2%) of the 16 *mefE*-positive isolates lacked *tetM*, indicating the absence of Tn*916*-like mobile genetic elements in these isolates. Importantly, 29 (19.2%) and 40 (26.5%) of the 151 isolates harboured the *folA* I100L mutation and *folP* insertion, respectively. We examined the types of transposons carrying the macrolide resistance genes by investigating the assembled contigs. Among the 134 isolates, the transposon structure was resolved in 126 because the relevant elements were captured in single, non-fragmented contigs. The most frequently detected transposon was Tn*3872* (*n*=42), followed by Tn*6002* (*n*=34) (Fig. S5). Additionally, five isolates carried a macrolide efflux genetic assembly in the absence of *tetM*. Transposons whose structures differed markedly from the reference sequences used in this study are shown in Figs S6–S8.

Among the 48 PEN-resistant isolates, the most prevalent PBP profile was *pbp1a*, *pbp2b:pbp2x*=13:175:43 (*n*=10), followed by 13:175:JP3 (*n*=7) and 4:7:7 (*n*=7) (Table S3). Among the 24 CTX- and 28 MEM-resistant isolates, the most prevalent PBP profiles were *pbp1a:pbp2b:pbp2x*=13:175:JP3 (*n*=7) and 13:175:43 (*n*=9).

We observed 16 multi-*β*-lactam resistant (resistant to PEN, CTX and MEM) isolates comprising 3 CCs, 63 (serotype 15A), 156 (serotype 23A, 23B or 35B) and 558 (serotype 35B), including 7 (43.8%) of non-IPD isolates.

Based on the ST and PBP profiles, we identified two serotype changes caused by recombination events. One was between the ST5242 serotype 23A and serotype 15C isolates with *pbp1a:pbp2b:pbp2x*=19:19:200. Another was between ST2754 serotypes 33B and 24B, with *pbp1a* type change between types 2 and 0.

Individual accession numbers for all isolates are listed in Table S4.

## Discussion

*S. pneumoniae* remains one of the most important pathogens in children, particularly infants and young children, even after the introduction of PCVs. This is because *S. pneumoniae* is highly competent and can change its serotype through genetic recombination, resulting in epidemiological changes in serotype distribution. Therefore, continuous surveillance studies are needed to develop new strategies for preventing pneumococcal diseases, including multivalent conjugate vaccines.

We started this study in March 2020, immediately after the onset of the COVID-19 pandemic. Similar to many other countries [39], the number of cases attributable to pneumococci in all populations decreased after the COVID-19 pandemic in Japan [4], resulting in a relatively small number of cases compared to our previous surveillance studies [9,10].

Compared with the results of a surveillance study conducted between 2015 and 2017, the percentage of isolates with PCV13 serotypes among IPD decreased from 9.2 to 0.8%. This decrease was largely due to a reduction in the number of cases caused by serotypes 19A and 1. During this study period, the most frequently detected serotype in patients with IPD was 15B/C, followed by 15A, 24B and 24F. In this study, 85.7% of the 15B/C isolates detected belonged to CC199 and GPSC4, of which 73.3% harboured the *pbp1a:pbp2b:pbp2x*=2:26:498 PBP profile. These 15B/C isolates were susceptible to *β*-lactam antibiotics; however, all strains showed resistance to macrolides (ERY) due to the presence of the *ermB* gene. Macrolide resistance in pneumococci may be due to their increasing empirical use in the treatment of pneumonia because of their broad-spectrum action against atypical pneumonia caused by *Mycoplasma pneumoniae*, *Chlamydia pneumoniae* and *Bordetella pertussis* [40]. Around 2010, excessive use of macrolides in Japan compared to other countries was reported [41]; macrolides accounted for a substantial proportion of oral antimicrobial consumption in Japan, representing 33.1% of total oral use in 2013. Accordingly, during this period, the prevalence of macrolide resistance among pneumococcal isolates detected in both IPD and non-IPD children was extremely high, at ~90% [9]. Since 2018, Japan has implemented a healthcare reimbursement scheme that allows medical facilities to claim a small financial incentive when antibiotics are not prescribed to children under 6 years of age in outpatient settings [42]. Reports on the impact of this policy on the antimicrobial susceptibility of bacterial pathogens, including *S. pneumoniae*, are awaited.

Serotype 15A has consistently been one of the most frequently detected serotypes since the start of a nationwide pneumococcal surveillance study in 2012 [9,10]. In this study and previous studies, most of the 15A strains detected in Japan belonged to CC63 and GPSC904;9 and tended to show resistance to *β*-lactam antibiotics, including MEM. Additionally, 15A isolates belonging to ST9084 were resistant to PEN, CTX, MEM and ERY [16]. Since 15A is not covered by PCV15 or PCV20, there is a strong possibility that it will become more selected in the future, potentially increasing its proportion among pneumococcal strains detected in Japan. Considering this background, close attention must be paid to future trends in the prevalence of 15A in Japan.

In this study, the prevalence of MEM resistance among IPD isolates was 14.3%, which is lower than the nationwide MEM resistance rate of 20.2% reported by JANIS in 2023. This discrepancy may be attributable to differences in the study populations, as the JANIS data encompass isolates obtained from individuals of all ages, including older adults, whereas the present study focused exclusively on paediatric isolates. This study was influenced by the COVID-19 pandemic and limited by a relatively small sample size, which may have increased the risk of bias. Nevertheless, the prevalence of MEM resistance among paediatric IPD isolates was 12.7% from 2015 to 2017, suggesting a potentially increasing trend warranting attention.

Moreover, the isolates derived from non-IPD cases exhibited significantly higher rates of resistance to PEN, CTX and MEM than those from IPD cases. This trend was also observed in our previous nationwide surveillance study conducted between 2015 and 2017 [10]. Younger children tend to have a significantly longer carriage duration of Penicillin-resistant *S. pneumoniae* (PRSP) [43], and the recent use of *β*-lactam antibiotics within approximately the past 1–6 months increases the likelihood of PRSP detection [44,45]. The relatively small number of non-IPD isolates in this study warrants careful consideration of the potential impact of sampling bias on the results. Nevertheless, in non-IPD patients, prior administration of antibiotics before sample collection may have selected for *β*-lactam-resistant pneumococci. Carefully monitoring whether such resistant pneumococci can cause IPD through capsular switching under vaccine-induced selective pressure is important.

Serotype 24F began to increase in detection primarily from patients with IPD in Japan around 2013; during the 2015–2017 surveillance period, it was the most frequently detected capsular type [10]. The 24F strains detected in Japan between 2012 and 2017 belonged to either ST2572 or its single-locus variant, ST5496 (GPSC106) [9,10]. However, in this study, we observed a sharp decline in cases of pneumococcal infections attributable to serotype 24F in 2021, the second year of the COVID-19 pandemic. Although this temporal trend suggests that these strains may have been affected directly or indirectly by the pandemic, we cannot exclude the possibility that the decline also reflects natural cyclical variations in serotype prevalence. Many studies have demonstrated the emergence and increase in patients with IPD by 24F after the introduction of PCV7 and/or 13, a few of which are antibiotic-resistant lineages [40,46, 47]. The serotype 24F isolates collected in this study were ST2572 (GPSC106) and ST162, a single-locus variant of ST156 (GPSC6), both of which were susceptible to PEN, CTX and MEM with *folA* mutation and/or *folP* insertion. According to the Global Pneumococcal Sequencing Project database on the PathogenWatch website [36], ST2572 isolates were submitted only from East Asian countries (Thailand, Myanmar and Hong Kong), all of which were serotype 23A, suggesting that this clone might have changed from serotype 24F in Japan and spread there. In contrast, serotype 24F-CC156 strains are prevalent in Europe and the USA [40,48], and their drug resistance profiles (i.e. susceptibility to PEN and EM and resistance to co-trimoxazole) are similar to those collected in this study. In our 2012–2017 surveillance study, the detection of 24F-ST162 was extremely rare, and only a single isolate was identified. In contrast, the present surveillance revealed eight 24F-ST162 isolates from patients with IPD. In addition, three isolates of 35B-ST156 were newly detected in both IPD and non-IPD cases, suggesting a possible increase in the prevalence of the CC156 clone. Serotype 35B-ST156 isolates had a PBP profile of *pbp1a:pbp2b:pbp2x*=4:12:7, while the European and current 24F-CC156 isolates in Japan had *pbp1a:pbp2b:pbp2x*=78:0:0, suggesting that the 24F-CC156 clone was likely imported from abroad around the end of the 2010s. In addition, we collected serotypes 23A, 23B and 35B-CC156, a few of which were likely to be resistant to *β*-lactams; therefore, we believe it is necessary to closely observe the trend of this clone and continuously monitor it.

The present study had some limitations. First, as the surveillance study was passive, it might not accurately reflect the nationwide epidemiology of paediatric pneumococcal diseases in Japan. The Japanese Ministry of Health, Labour and Welfare reported 279 cases of IPD in children aged 0–4 years in 2020 compared to 43 cases included in our study. Accordingly, our surveillance may have captured ~one-sixth of the total paediatric IPD cases in Japan; however, 2020 marked the beginning of the COVID-19 pandemic, and whether the reporting system functioned properly during this period is uncertain. In addition, severe cases were more likely to be reported, whereas milder cases were less likely. This preferential inclusion of severe cases may have influenced the observed serotype distribution. Nevertheless, because IPD cases were reported in 54 clinics/hospitals in 30 of the 47 prefectures, we consider that substantial geographic bias was unlikely. Second, because only 25 non-IPD isolates were included, the observed distribution of serotypes and antimicrobial susceptibilities was likely strongly influenced by sampling bias. Finally, as the present study was not population-based, it was not possible to assess increasing or decreasing trends of IPD cases.

In Japan, PCV15 was approved in April 2024, and it replaced PCV13 for routine paediatric vaccinations. In addition, PCV20 was introduced in October 2024. Our serotype prevalence data showed that the PCV15 and PCV20 vaccines have the potential to cover an additional 12.7 and 42.1% of current IPD isolates, respectively. However, similar to the case following the previous introduction of new multivalent PCVs, capsular switching is expected to lead to the emergence of new non-vaccine serotype clones, gradually reducing the number of isolates with serotypes included in PCVs. Therefore, we believe that it is important to monitor seroprevalence in addition to antimicrobial susceptibility and genomic characteristics.

Furthermore, this study demonstrated the persistently high prevalence of macrolide resistance since the 2010s, as well as the higher frequency of *β*-lactam resistance among non-IPD isolates compared with IPD isolates. Although overuse of macrolides is the most plausible explanation for the high prevalence of macrolide resistance, the behaviour of macrolide resistance genes located on transposons under macrolide exposure remains unclear. Because *β*-lactam resistance arises from recombination events in PBP regions through natural transformation, the frequency of *β*-lactam resistance-associated PBPs within the donor gene pool is expected to influence the future epidemiology of *β*-lactam resistance. Taken together, clarifying the epidemiological impact of nationwide antimicrobial stewardship policies on pneumococcal resistance, as well as the dynamics of transposons carrying macrolide resistance genes in pneumococcal populations under macrolide exposure, and the relationship between the content of the gene pool revealed by metagenomics and the emergence of *β*-lactam-resistant pneumococci is important.

## Supplementary material

10.1099/jmm.0.002105 Uncited Fig. S1.

10.1099/jmm.0.002105 Uncited Fig. S2.

## Abbreviations

CC: clonal complex
CLSI: Clinical and Laboratory Standards Institute
CTX: cefotaxime
ERY: erythromycin
GPSC: Global Pneumococcla Sequence Cluster
IPD: invasive pneumococcal disease
JANIS: Japan Nosocomial Infections Surveillance
LRT: likelihood ratio test
MEM: meropenem
MLST: Multilocus sequence typing
OR: odds ratio
PBP: penicillin-binding protein
PCV: pneumococcal conjugate vaccine
PEN: penicillin
SNPs: Single Nucleotide Polymorphisms
ST: sequence type
