# Supplementary material for: Serotype distribution and antimicrobial resistance of Streptococcus pneumoniae in paediatric patients in Japan (2020–2023)
Source: J Med Microbiol. 2025 Dec 12;74(12):002105. doi: 10.1099/jmm.0.002105 (PMC12699485; doi:10.1099/jmm.0.002105)
Supplement: Uncited Fig. S1. [file jmm-74-02105-s001.pdf]

Title: Serotype Distribution and Antimicrobial Resistance of Streptococcus pneumoniae in Pediatric Patients in Japan (2020–2023).

Supplementary materials

Table S1. Antimicrobial resistance rates of IPD and non-IPD isolates collected in Japan between 2020-2023.

IPD or non-IPD	Antibiotic	No. of isolates (%)		*P* value (IPD vs non-IPD)*
		Susceptible	Resistance	
IPD	PEN	93 (73.8)	33 (26.2)	
Non-IPD	PEN	10 (40.0)	15 (60.0)	0.0071
IPD	CTX	112 (88.9)	14 (11.1)	
Non-IPD	CTX	15 (60.0)	10 (40.0)	0.0047
IPD	MEM	108 (85.7)	18 (14.3)	
Non-IPD	MEM	15 (60.0)	10 (40.0)	0.034
IPD	EM	16 (12.7)	110 (87.3)	
Non-IPD	EM	1 (4.0)	24 (96.0)	1.0

**P* values were calculated by Fisher's exact test with Bonferroni correction.

Table S2. Prevalence of sequence types and clonal complexes among isolates derived from IPD and non-IPD patients in Japan, 2020-2022. *

Serotype, no. of isolates collected in years 2020:2021:2022 (from non-IPD cases)	CCs or STs[a] (n)	GPSC (n)	*ermB*/*mefE* (n)	No. of isolates with *folA* substitution (%)	No. of isolates with *folP* insertion (%)	*tetM* (n)	Pili (n)
3, 0:1(1):0	180 (1)	12 (1)	*ermB* (1)	0 (0)	0 (0)	1 (100)	None (1)
6C, 1:0:0	5832 (1)	5 (1)	*mefE* (1)	0 (0)	1 (100)	1 (100)	None (1)
7C, 2:0:0	2758 (2)	248 (2)	*ermB* (2)	2 (100)	2 (100)	2 (100)	None (2)
10A, 1(1):9(2):6(1)	1263 (6)	NA (5), 634 (1)	*ermB* (3), None (3)	0 (0)	2 (33.3)	3 (50)	None (6)
	5236 (10)	NA (10)	*ermB* (10)	0 (0)	0 (0)	10 (100)	None (10)
11A, 1:1:0	99 (2)	73 (2)	*ermB* + *mefE* (1), *mefE* (1)	0 (0)	0 (0)	2 (100)	None (2)
12F, 3:2:0	6945 (5)	334 (5)	*ermB* (5)	2 (40)	2 (40)	5 (100)	None (5)
15A, 7(3):7(2):6(1)	63 (19)	904;9 (19)	*ermB* (19)	0 (0)	0 (0)	19 (100)	None (19)
	5242 (1)	5 (1)	*ermB* (1)	0 (0)	0 (0)	1 (100)	None (1)
15B, 2(1):7:10(1)	199 (15)	4 (15)	*ermB* (15)	0 (0)	0 (0)	15 (100)	None (15)
	3111 (1)	932 (1)	*ermB* + *mefE* (1)	0 (0)	0 (0)	1 (100)	None (1)
	5242 (2)	5 (2)	*ermB* (2)	0 (0)	0 (0)	2 (100)	None (2)
	18745 (1)	4 (1)	*ermB* (1)	0 (0)	0 (0)	1 (100)	None (1)
15C, 5(1):10(3):1	199 (15)	4 (15)	*ermB* (15)	0 (0)	0 (0)	15 (100)	None (15)
	5242 (1)	5 (1)	*ermB* (1)	0 (0)	0 (0)	1 (100)	None (1)
16F, 0:1:0	13304 (1)	NA (1)	None (1)	0 (0)	0 (0)	0 (0)	None (1)
19A, 1:0:0	3111 (1)	932 (1)	*ermB* + *mefE* (1)	0 (0)	0 (0)	1 (100)	Pili1 (1)
20, 0:0:1	4745 (1)	43 (1)	*ermB* (1)	0 (0)	1 (100)	1 (100)	None (1)
21, 1(1):0:0	1233 (1)	67 (1)	*mefE* (1)	0 (0)	0 (0)	1 (100)	None (1)
22F, 2:2:1	433 (5)	19 (5)	*ermB* (3), mef*E* (1), None (1)	0 (0)	0 (0)	3 (60)	None (5)
23A, 3 (1):0:1	156 (1)	6 (1)	*ermB* (1)	1 (100)	0 (0)	1 (100)	Pili1 (1)

	5242 (3)	5 (3)	*ermB* (3)	0 (0)	0 (0)	3 (100)	None (3)
23B, 0:1(1):0	156 (100)	6 (1)	*ermB* (1)	1 (100)	0 (0)	1 (100)	Pili1 (1)
24B, 7:2:4	156 (1)	6 (1)	*ermB* (1)	1 (100)	1 (100)	1 (100)	Pili1 (1)
	2572 (2)	106 (2)	*ermB* (2)	0 (0)	2 (100)	2 (100)	None (2)
	2754 (10)	230 (10)	*ermB* (10)	10 (100)	10 (100)	10 (100)	None (10)
24F, 10:1:1	156 (7)	6 (7)	*ermB* (1)	7 (100)	7 (100)	1 (14)	Pili1 (7)
	2572 (5)	106 (5)	*ermB* (5)	0 (0)	5 (100)	5 (100)	None (0)
28F, 0:1:0	546 (1)	191 (1)	None (1)	0 (0)	0 (0)	0 (0)	None (1)
33B, 0:0:1(1)	2754 (100)	230	*ermB* (1)	1 (100)	1 (100)	1 (100)	None (1)
33F, 5(1):5:2	673 (3)	3 (3)	None (3)	0 (0)	0 (0)	0 (0)	None (3)
	717 (9)	3 (9)	*ermB* (9)	0 (0)	0 (0)	9 (100)	None (9)
34, 0:1:1	3116 (2)	45 (2)	*mefE* (1), None (1)	0 (0)	1 (50)	1 (50)	None (2)
35B, 0:6(1):6(2)	156 (3)	6 (3)	*mefE* (3)	3 (100)	3 (100)	0 (0)	Pili1 (3)
	558 (8)	59 (8)	*mefE* (8)	0 (0)	0 (0)	7 (88)	Pili1 (3)
	2755 (1)	186 (81)	*ermB* (1)	1 (100)	1 (100)	1 (100)	None (1)
35F, 0:0:1	8296 (1)	36 (1)	None (1)	0 (0)	0 (0)	0 (0)	None (1)
NT, 1:0:0	9366 (100)	NA	*ermB* (1)	0 (0)	1 (100)	1 (100)	None (1)

*CCs were defined in agreement with five of the seven loci. If there were no different STs sharing five or six alleles at the locus, the ST number was listed. We searched for I100L substitutions and D92R in *folA*, and only I100L substitutions were detected.

Table S3. PBP profile in each serotype and the association with the beta β-lactum resistance.

Serotype, no. of isolates collected in years 2020:2021:2022 (from non-IPD cases)	CCs or STs[a] (n)	GPSC (n)	PBP profile (*pbp1a*:*pbp2b*:*pbp2x*) (n)	PEN:CTX:MEM (n)
3, 0:1(1):0	180 (1)	12 (1)	2:0:168 (1)	S:S:S (1)
6C, 1:0:0	5832 (1)	5 (1)	0:86:80 (1)	R:S:S (1)
7C, 2:0:0	2758 (2)	248 (2)	2:4:375 (2)	S:S:S (2)
10A, 1(1):9(2):6(1)	5236 (10)	NA (10)	2:0:114 (9)	S:S:S (9)
			2:0:0 (1)	S:S:S (1)
	1263 (6)	NA (5), 634 (1)	120:4:73 (2)	R:R:S (2)
			2:4:100 (1)	S:S:S (1)
			2:4:JP35 (1)	S:S:S (1)
			8:4:JP25 (1)	R:I:S (1)
			8:4:JP79 (1)	S:S:S (1)
11A, 1:1:0	99 (2)	73 (2)	23:4:JP49 (1)	S:S:S (1)
			23:4:0 (1)	S:S:S (1)
12F, 3:2:0	6945 (5)	334 (5)	37:4:23 (3)	S:S:S (3)
			37:278:375 (2)	S:S:S (2)
15A, 7(3):7(2):6(1)	63 (19)	904;9 (19)	13:175:43 (10)	R:S:I (8)
				R:S:S (1)
				R:I:I (1)
			13:175:JP3 (7)	R:R:I (6)
				R:R:R (1)
			13:175:JP84 (1)	R:R:I (1)
			24:27:43 (1)	R:S:S (1)
	5242 (1)	5 (1)	19:1:JP53 (1)	R:S:S (1)
15B, 2(1):7:10(1)	199 (15)	4 (15)	2:26:498 (12)	S:S:S (12)

			13:26:498 (1)	R:S:S (1)
			2:0:JP55 (1)	S:I:S (1)
			7:0:JP2	S:S:S (1)
	5242 (2)	5 (2)	19:1:200 (1)	R:S:S (1)
			19:1:498 (1)	R:S:S (1)
	3111 (1)	932 (1)	13:24:JP85 (1)	R:S:S (1)
	18745 (1)	4 (1)	2:26:498 (1)	S:S:S (1)
15C, 5(1):10(3):1	199 (15)	4 (15)	2:26:498 (10)	S:S:S (10)
			13:0:47 (1)	R:I:S (1)
			13:0:JP80 (1)	R:I:S (1)
			13:16:JP82 (1)	R:R:S (1)
			2:0:JP24 (1)	S:S:S (1)
	5242 (1)	5 (1)	JP22:0:JP83 (1)	S:S:S (1)
16F, 0:1:0	13304 (1)	NA (1)	0:0:2 (1)	S:S:S (1)
19A, 1:0:0	3111 (1)	932 (1)	2:0:112 (1)	S:S:S (1)
20, 0:0:1	4745 (1)	43 (1)	0:4:23 (1)	S:S:S (1)
21, 1(1):0:0	1233 (1)	67 (1)	23:2:111 (1)	S:S:S (1)
22F, 2:2:1	433 (5)	19 (5)	1:2:8 (3)	S:S:S (3)
			1:2:2 (1)	S:S:S (1)
			JP23:2:8 (1)	S:S:S (1)
23A, 3 (1):0:1	5242 (3)	5 (3)	19:1:200 (2)	R:S:S (2)
			19:1:24 (1)	R:S:S (2)
	156 (1)	6 (1)	15:11:299 (1)	R:R:R (1)
23B, 0:1(1):0	156 (1)	6 (1)	15:11:299 (1)	R:R:R (1)
24B, 7:2:4	2754 (10)	230 (10)	2:0:JP38 (19)	S:S:S (10)
	2572 (2)	106 (2)	2:0:2 (2)	S:S:S (2)
	156 (1)	6 (1)	2:0:363 (1)	S:S:S (1)
24F, 10:1:1	156 (7)	6 (7)	78:0:0 (6)	S:S:S (6)
			2:0:363 (1)	S:S:S (1)

	2572 (5)	106 (5)	2:0:2 (5)	S:S:S (5)
28F, 0:1:0	546 (1)	191 (1)	2:2:2 (1)	S:S:S (1)
33B, 0:0:1(1)	2754 (1)	230 (1)	0:0:JP38 (1)	S:S:S (1)
33F, 5(1):5:2	717 (9)	3 (9)	2:0:6 (7)	S:S:S (7)
			2:0:200 (1)	S:S:S (1)
			2:0:JP81 (1)	S:S:S (1)
	673 (3)	3 (3)	0:0:3 (3)	S:S:S (3)
34, 0:1:1	3116 (2)	45 (2)	0:0:2 (1)	S:S:S (1)
			0:0:JP36 (1)	S:S:S (1)
35B, 0:6(1):6(2)	558 (8)	59 (8)	4:7:7 (8)	S:S:S (1)
				R:S:I (3)
				R:I:I (4)
	156 (3)	6 (3)	4:12:7 (3)	R:S:S (1)
				R:S:I (1)
				R:I:I (1)
	2755 (1)	186 (1)	0:0:JP34 (1)	S:S:S (1)
35F, 0:0:1	8296 (1)	36 (1)	0:0:3 (1)	S:S:S (1)
NT, 1:0:0	9366 (1)	NA	17:31:JP86 (1)	R:R:S (1)

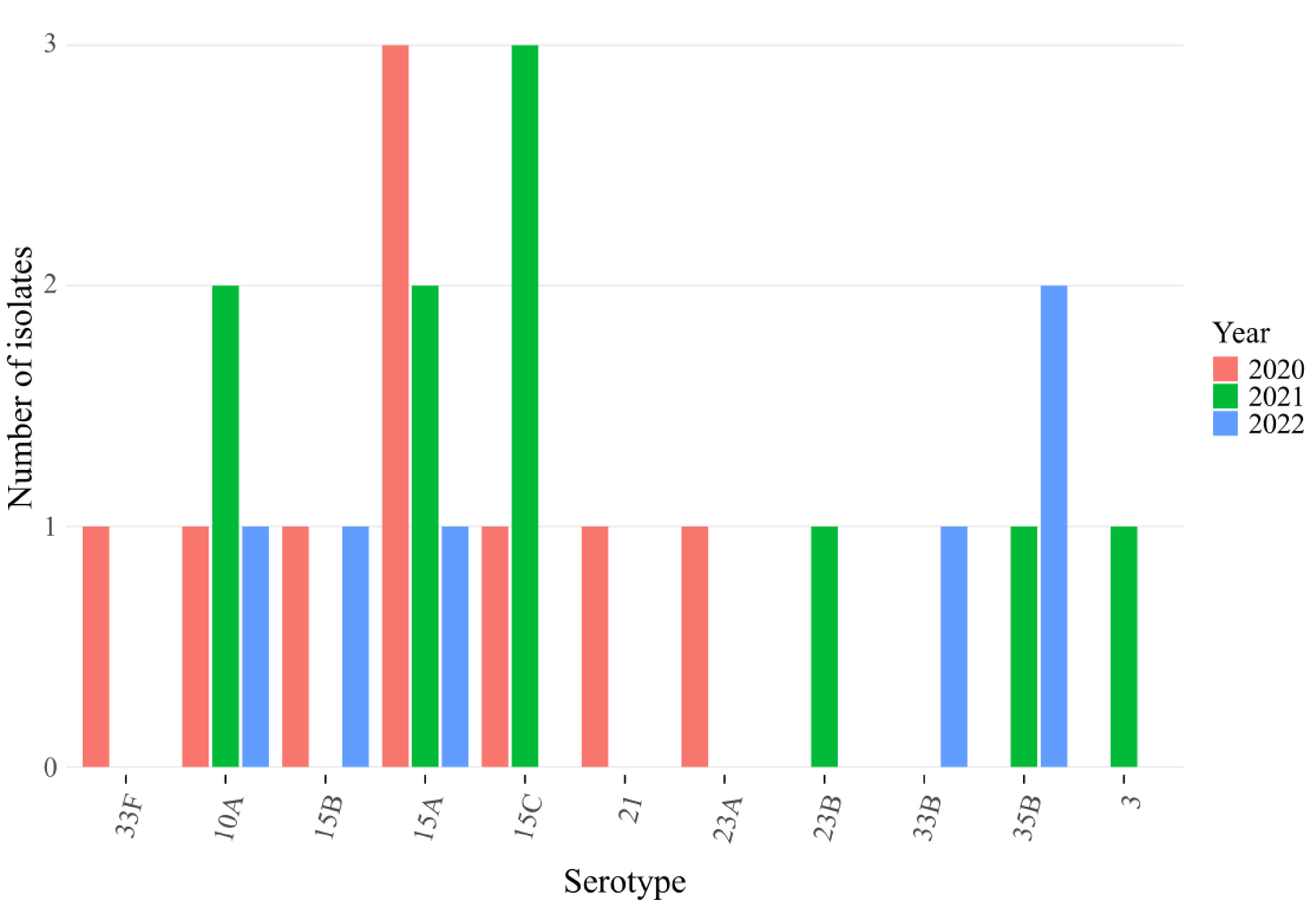

Figure S1. Seroprevalence of pediatric noninvasive pneumococcal disease between 2020 and 2022 in Japan

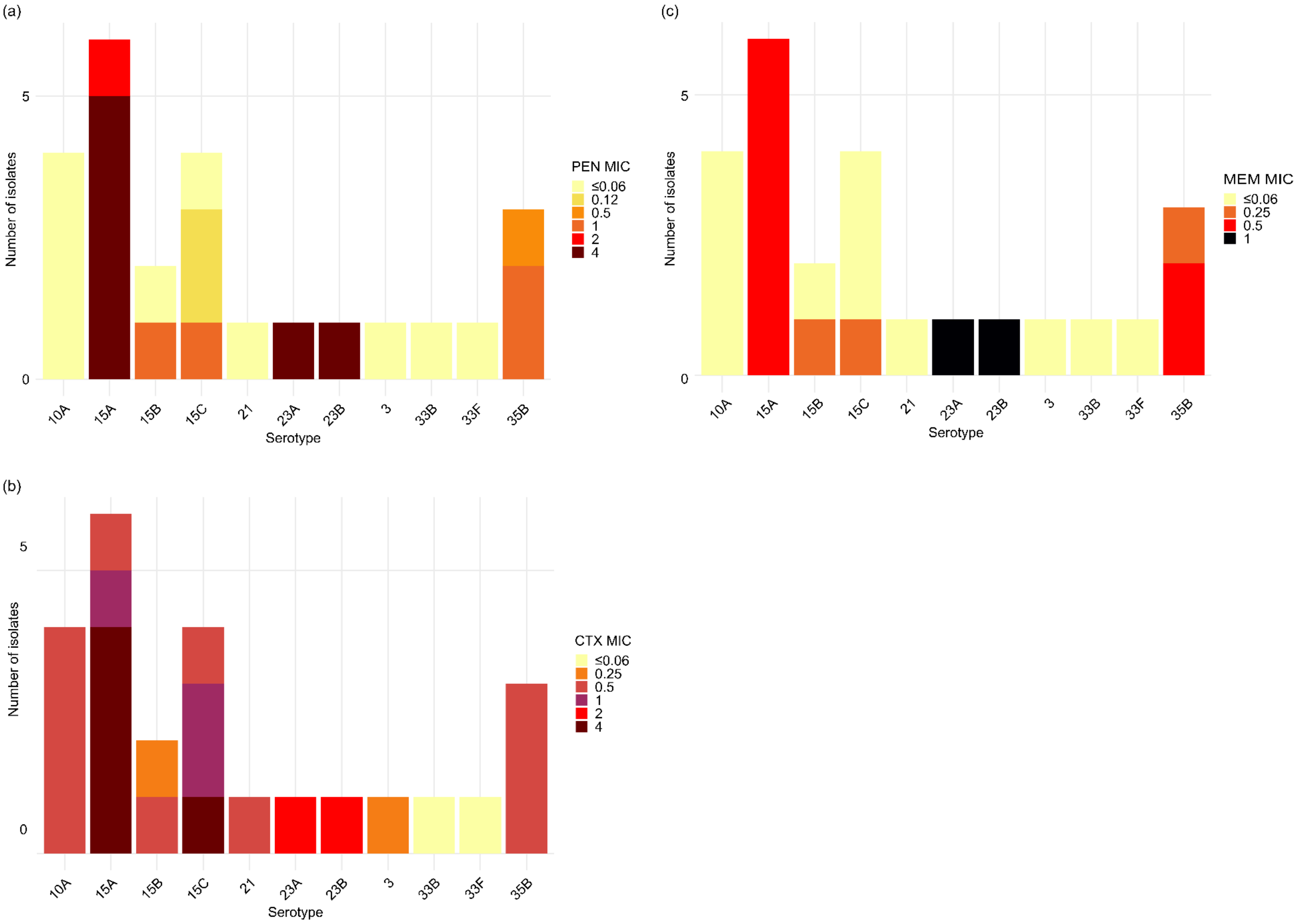

Figure S2. Minimum inhibitory concentration (MIC) distribution of penicillin, cefotaxime, and meropenem in each non-IPD pneumococcal serotype in Japan. PEN, penicillin; CTX, cefotaxime; MEM, meropenem.

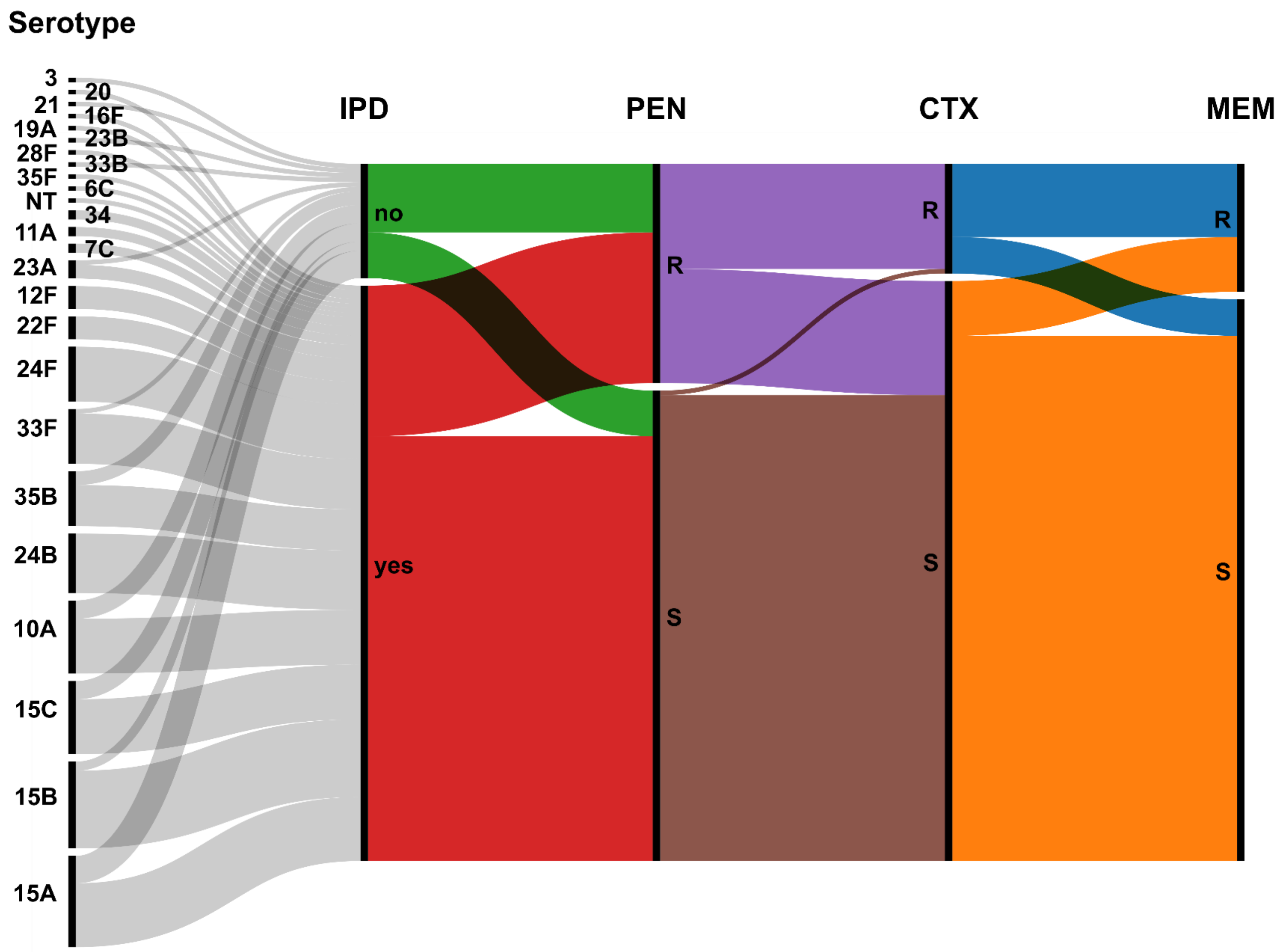

Figure S3. Alluvial Diagram showing the relationships between pneumococcal serotypes and β-lactam resistance of IPD and non-IPD isolates. Each flow represents the number of isolates showing the CLSI resistance categories of each antibiotic.

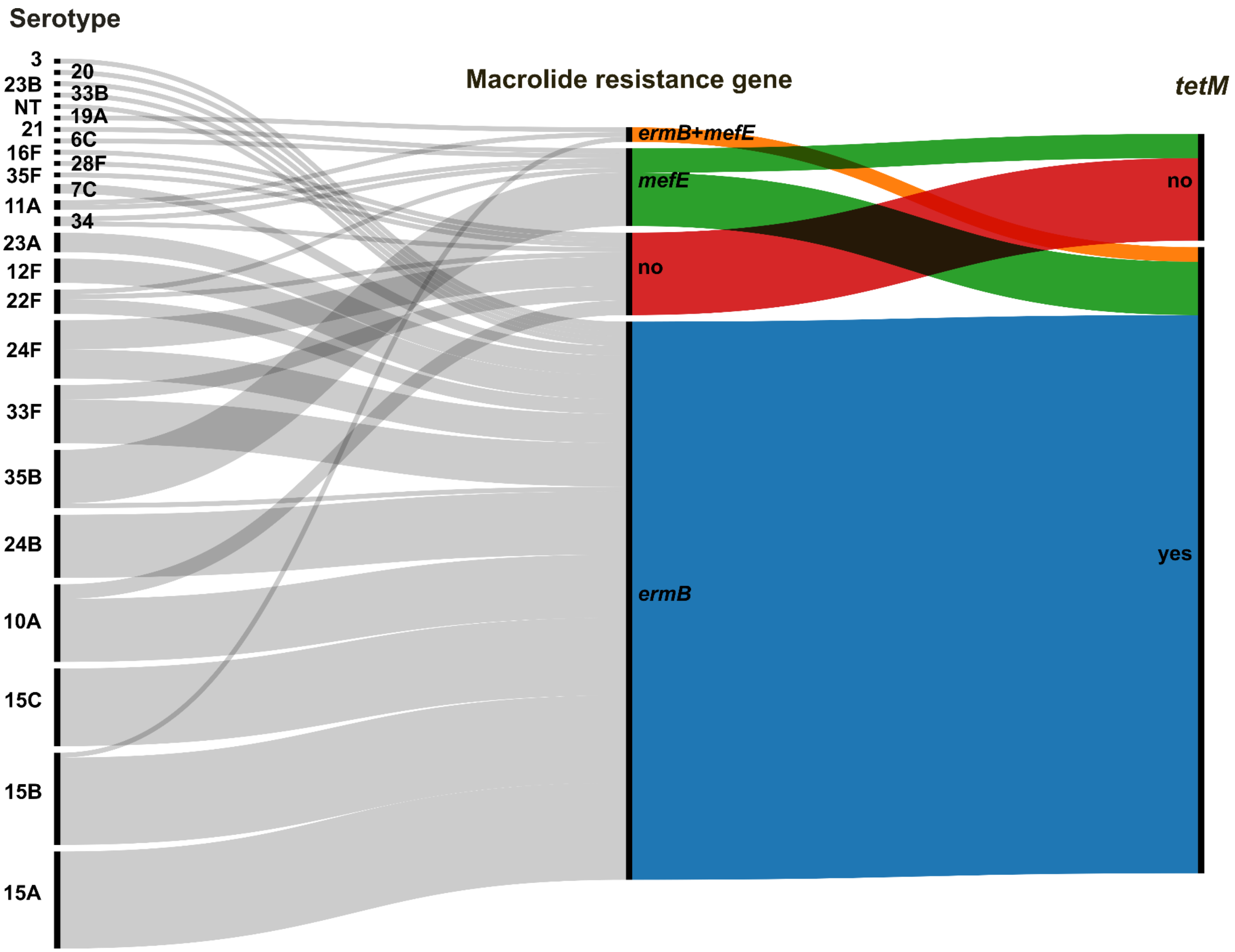

Figure S4. Alluvial Diagram showing the relationships between pneumococcal serotypes, macrolide resistance genes (*mefE* and/or *ermB*), and the presence of the tetracycline resistance gene *tetM* of IPD and non-IPD isolates. Each flow represents the number of isolates carrying the indicated genetic determinants.

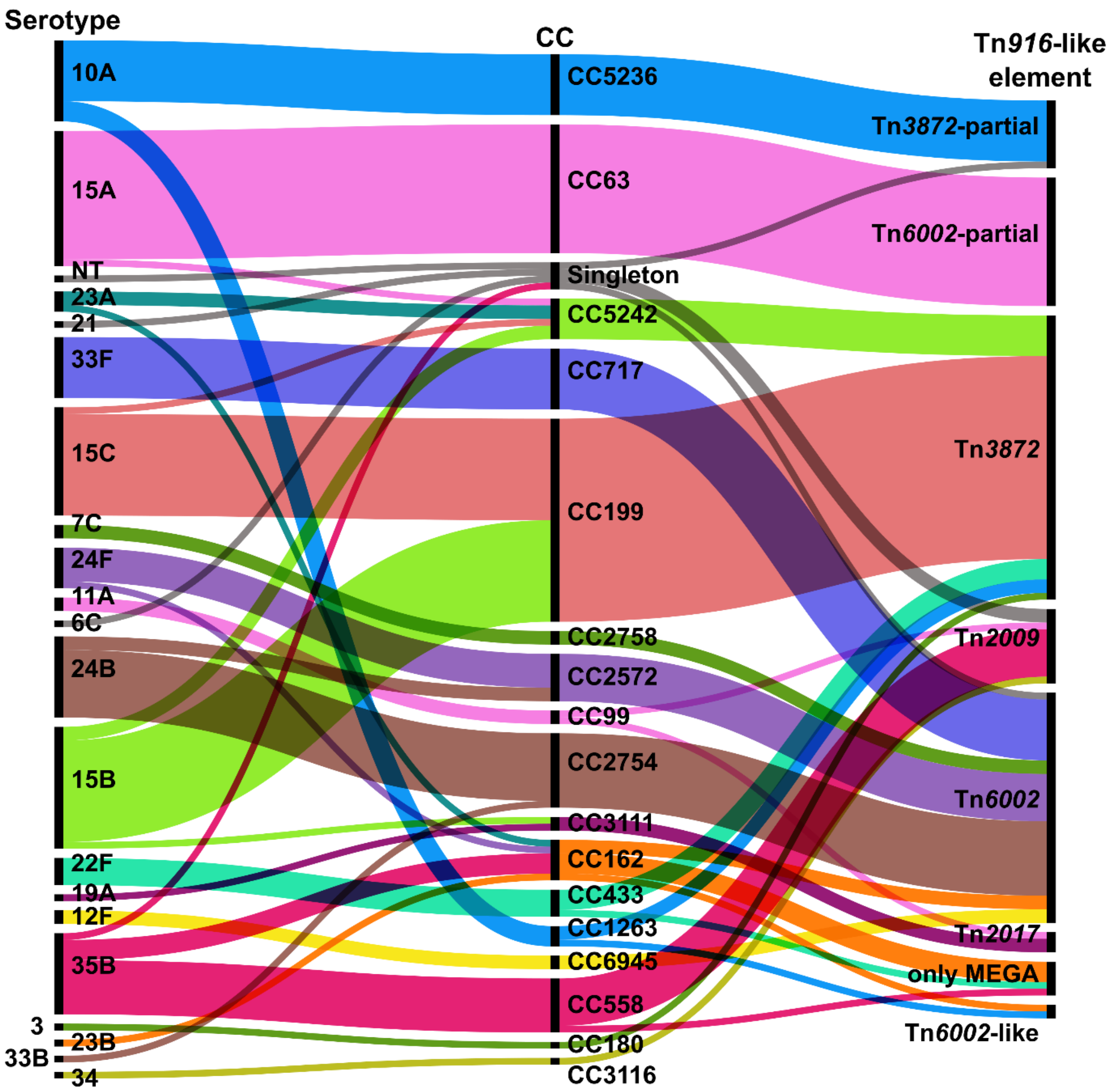

Figure S5. Alluvial Diagram showing the relationships between pneumococcal serotypes with macrolide resistance genes (i.e. *ermB* and/or *mefE*), clonal complex (CC) and Tn*916*-like elements. Each flow represents the number of isolates carrying the transposon. Five isolates had only macrolide efflux genetic assembly (MEGA) without Tn*916*-like element. Tn*3872*-partial and Tn*6002*-partial refer to structures in which the respective transposons are truncated and integrated into the chromosome. Details of these structures are provided in Figures S6 and S7.

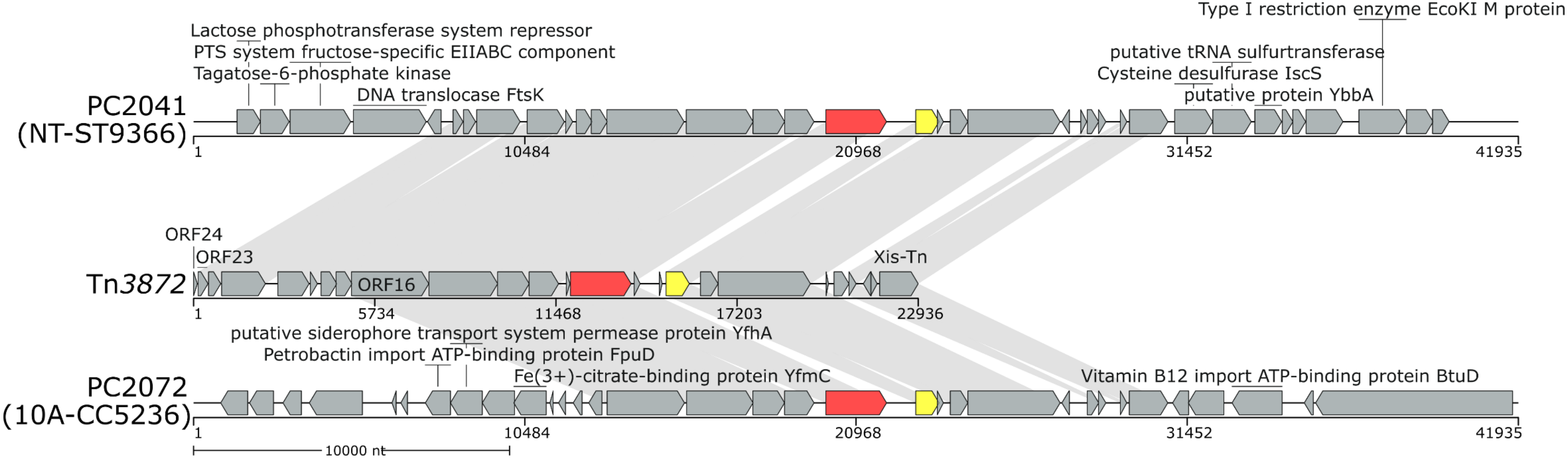

Figure S6. Structures of truncated Tn*3872* detected in serotype 10A-CC5236 isolates. The genes highlighted in red and yellow indicate *tetM* and *ermB*, respectively. In 10A-CC5236 (represented by strain PC2072), the upstream structure of ORF16 in the reference sequence was missing. PC2041, identified as NT (non-typeable)-ST9366, also harbored a truncated Tn*3872* similar to 10A-CC5236; however, its structure was preserved up to ORF23, with only ORF24 being deleted. The genes highlighted in red and yellow indicate *tetM* and *ermB*, respectively. The sequence of Tn*3872* was obtained from the NCBI database (accession number OP715845.1).

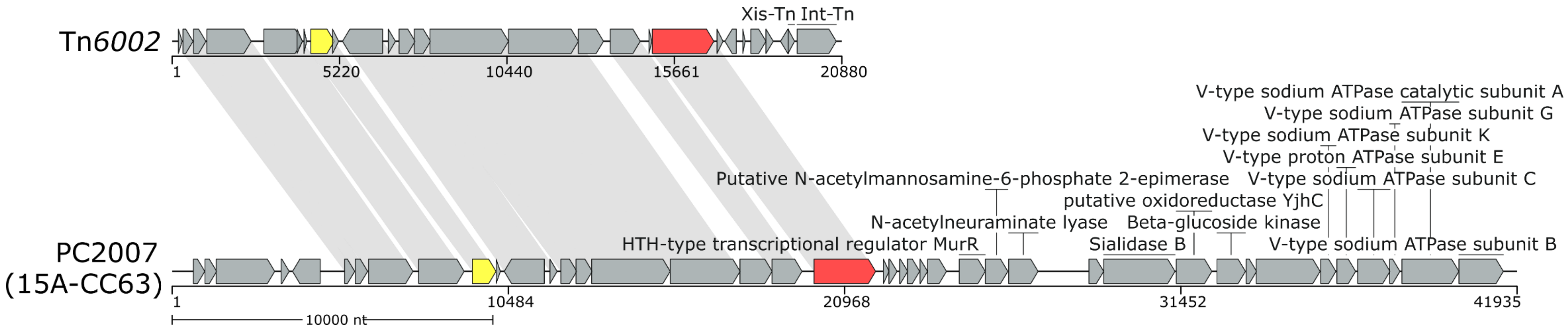

Figure S7. Structures of truncated Tn*6002* detected in serotype 15A-CC63 isolates. The genes highlighted in red and yellow indicate *tetM* and *ermB*, respectively. In 15A-CC63 (represented by strain PC2007), the downstream structure of *tetM* in the reference sequence was missing. The sequence of Tn*6002* was obtained from the NCBI database (accession number AY898750.1).

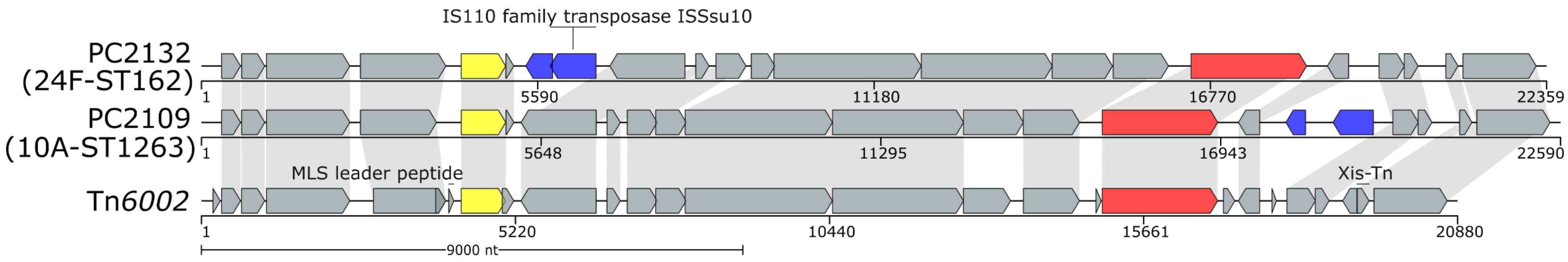

Figure S8. Structures of Tn*6002*-like elements that were detected in this study. The genes highlighted in red and yellow indicate *tetM* and *ermB*, respectively. The genes highlighted blue were inserted genes that were not associated with antimicrobial resistance. The sequence of Tn*6002* was obtained from the NCBI database (accession number AY898750.1).
